# Pericardial metastases from BRCA1-associated peritoneal cancer. Case report

**DOI:** 10.1186/1897-4287-13-S1-A17

**Published:** 2015-09-09

**Authors:** Anna Abramowska, Dariusz Godlewski

**Affiliations:** 1 Genetics Clinic, Centre of Cancer Epidemiology and Prevention, Poznan, Poland

## 

Women with BRCA1 gene mutations have about 30 to 40 times higher risk of ovarian cancer compared to the population risk (the lifetime risk of BRCA1-dependent ovarian cancer is about 40 percent). Because of the high risk BRCA1 mutations carriers at the age of about 40 are recommended prophylactic salpingo-oophorectomy (after completion of reproductive plans). After the surgery there is still a risk of peritoneal cancer which histologically resembles ovarian cancer, but it is much lower than the risk of ovarian cancer before salpingo-oophorectomy: the cumulative risk of developing cancer of the peritoneum is about 4 percent during 20 years from the removal of ovaries and fallopian tubes.

We are going to present a case of long lasting diagnosis of pericardial metastases from BRCA1-associated peritoneal cancer.

